# Neurite orientation dispersion and density imaging of white matter microstructure in sensory processing dysfunction with versus without comorbid ADHD

**DOI:** 10.3389/fnins.2023.1136424

**Published:** 2023-07-10

**Authors:** Ian T. Mark, Jamie Wren-Jarvis, Jaclyn Xiao, Lanya T. Cai, Shalin Parekh, Ioanna Bourla, Maia C. Lazerwitz, Mikaela A. Rowe, Elysa J. Marco, Pratik Mukherjee

**Affiliations:** ^1^Department of Radiology and Biomedical Imaging, University of California – San Francisco, San Francisco, CA, United States; ^2^Cortica Healthcare, San Rafael, CA, United States

**Keywords:** sensory processing dysfunction, attention deficit and hyperactivity disorder, diffusion MRI, white matter microstructure, neurite orientation dispersion and density imaging

## Abstract

**Introduction:**

Sensory Processing Dysfunction (SPD) is common yet understudied, affecting up to one in six children with 40% experiencing co-occurring challenges with attention. The neural architecture of SPD with Attention Deficit and Hyperactivity Disorder (ADHD) (SPD+ADHD) versus SPD without ADHD (SPD-ADHD) has yet to be explored in diffusion tensor imaging (DTI) and Neurite Orientation Dispersion and Density Imaging (NODDI) has yet to be examined.

**Methods:**

The present study computed DTI and NODDI biophysical model parameter maps of one hundred children with SPD. Global, regional and voxel-level white matter tract measures were analyzed and compared between SPD+ADHD and SPD-ADHD groups.

**Results:**

SPD+ADHD children had global WM Fractional Anisotropy (FA) and Neurite Density Index (NDI) that trended lower than SPD-ADHD children, primarily in boys only. Data-driven voxelwise and WM tract-based analysis revealed statistically significant decreases of NDI in boys with SPD+ADHD compared to those with SPD-ADHD, primarily in projection tracts of the internal capsule and commissural fibers of the splenium of the corpus callosum.

**Conclusion:**

We conclude that WM microstructure is more delayed/disrupted in boys with SPD+ADHD compared to SPD-ADHD, with NODDI showing a larger effect than DTI. This may represent the combined WM pathology of SPD and ADHD, or it may result from a greater degree of SPD WM pathology causing the development of ADHD.

## Introduction

Sensory Processing Dysfunction (SPD) refers to a clinical deficit in the ability to modulate, discriminate, or create an organized response to sensory information, affecting up to 16% of children (Ben-Sasson et al., 2009). Due to the disruptions in sensory processing, children with SPD may demonstrate atypical or delayed intellectual, language, or motor milestones (May-Benson et al., 2009).

Of children with SPD, approximately 40% will also meet research criteria for attention-deficit/hyperactivity disorder (ADHD) (Koziol and Budding, 2012). ADHD is characterized by pervasive and disabling symptoms of inattention, hyperactivity, and impulsivity, as defined by the Diagnostic and Statistical Manual of Mental Diseases (DSM-5), and affects 5–8% of school children (Faraone et al., 2003; American Psychiatric Association, 2013). However, the rate of ADHD in SPD is almost ten-fold higher. While there are overlapping features of ADHD and SPD, they may represent two distinct dimensions that can coexist with unique neurobiological properties (Miller et al., 2012). SPD in the context of autism and ADHD has been associated with mental health and behavioral challenges including anxiety, depression, academic difficulties, and disruptive behaviors (Mangeot et al., 2001; Sanz-Cervera et al., 2017; MacLennan et al., 2021; Rossow et al., 2021, 2022). Since SPD is a global cluster of sensory discrimination, modulation and sensorimotor challenges that often co-occurs with attention challenges. It is likely that these information processing functions have both shared and unique aspects of their underlying neural mechanisms. Teasing apart these neural underpinnings will shed light on how these two dimensions thought to be distinct might interrelate (Miller et al., 2012).

Quantitative neuroimaging studies found no differences in gray or white matter (WM) volumes between children with SPD and the typically developing childhood cohort, indicating that the pathology should be sought in microstructural changes (Owen et al., 2013; Brandes-Aitken et al., 2018b; Narayan et al., 2021). Diffusion magnetic resonance imaging (dMRI) research has shown that diffusion tensor imaging (DTI) -derived fractional anisotropy (FA) in the superior corona radiata uniquely correlates with cognitive control performance in subjects with SPD (Brandes-Aitken et al., 2018a). However, no prior work has examined the WM microstructure in SPD with ADHD (SPD+ADHD) compared to SPD without ADHD (SPD-ADHD). SPD+ADHD children have been shown to have reduced midline frontal theta activity on electroencephalography (EEG), a marker of attention abilities captured in real-time (Anguera et al., 2017). The midline frontal theta difference is thought to emanate from the dorsal anterior cingulate and adjacent medial prefrontal cortex, a region that would be expected to be implicated *a priori* due to its known importance for impulse control (Ishii et al., 2014).

Although DTI is a useful tool for studying brain development, it represents only a basic statistical description of water diffusion within a voxel. DTI is typically acquired at a single relatively low diffusion-weighting factor (b value) representing only a single spherical shell in q-space. The assumption of Gaussian diffusion that underpins the DTI model breaks down at b values in excess of 1,000 s/mm^2^, whereas the investigation of restricted and strongly hindered diffusion, such as within the intracellular space, requires higher diffusion-weighting factors. Therefore, common DTI measures, such as fractional anisotropy (FA), mean diffusivity (MD), axial diffusivity (AD), and radial diffusivity (RD) are not specific to the underlying microstructural features of axons and dendrites (collectively called neurites) and often lack the specificity to differentiate between intracellular and extracellular disruption (Pierpaoli and Basser, 1996; Wheeler-Kingshott and Cercignani, 2009; De Santis et al., 2014). In contrast to DTI, Neurite Orientation and Dispersion Density Imaging (NODDI) is a multi-compartment biophysical model of brain microstructure that computes the non-collinear properties of neurite Orientation Dispersion Index (ODI) and Neurite Density Index (NDI), corresponding to the degree of incoherence in fiber orientations and to the intracellular volume fraction within each imaging voxel, respectively. NODDI employs a tissue model that distinguishes three types of microstructural environment: restricted intracellular compartment modeled with orientation dispersion using a Watson distribution; (May-Benson et al., 2009) extracellular compartment with Gaussian anisotropically hindered diffusion and (Koziol and Budding, 2012) cerebrospinal fluid (CSF) compartment with freely isotropic diffusion. NODDI allows *in vivo* estimation of NDI, ODI, and free water fraction (FISO) (Fukutomi et al., 2019). NDI is typically considered to be the most sensitive NODDI metric for white matter microstructure (Mah et al., 2017).

Our study is the first to examine the neural architecture of SPD+ADHD versus SPD-ADHD using either DTI or NODDI. Prior studies have shown that FA and NDI are the most sensitive DTI and NODDI metrics in brain development and WM diseases, with rising FA and NDI values during childhood WM maturation and decreased FA and NDI in most forms of WM pathology (Mukherjee et al., 2001, 2002; Mukherjee and McKinstry, 2006; Chang et al., 2015a,b). Previous work in SPD patients has shown reduced FA in posterior projection and commissural tracts of the bilateral posterior thalamic radiation and splenium of the corpus callosum, as well as the posterior limb of internal capsule (Chang et al., 2015a,b). White matter abnormalities in ADHD have been studied using DTI, although showing heterogeneous results with a lack of consensus in the literature. A meta-analysis of tract-based spatial statistics (TBSS) hypothesizes that the fronto-striatal-cerebellar circuit plays a crucial role in the pathophysiology of ADHD (Chen et al., 2016). We hypothesize that the SPD+ADHD cohort will have globally lower FA and NDI than the SPD-ADHD group, that this difference may be more robust for neural tracts previously implicated in attention, and that the intracellular component as measured by NDI may show a stronger effect than the basic tract anisotropy as measured by FA.

## Materials and methods

### Participants

We prospectively enrolled and evaluated children between 8–12 years of age at a community neurodevelopmental clinic. The research protocol of the present study was approved by the institutional review board at our medical center with written informed consent obtained from the parents or legal guardians and assent obtained from the study participants. Exclusion from the study is based on the following criteria:

- Nonverbal Index ≤70 on the Wechsler Intelligence Scale for Children, Fifth Edition
- < 1 ‘Yes’ or < 2 ‘Maybe / A Little’ responses on the ESSENCE-Q-REV parent questionnaire for neurodevelopmental concerns
- Caregiver(s) unable to complete intake forms
- *In utero* toxin exposure
- Gestational age < 32 weeks or intrauterine growth restriction (birth weight < 1,500 grams)
- Hearing or visual impairment
- Additional medical/neurologic condition, including active epilepsy, malignancy, or known brain injury/malformation

All participants were assessed for SPD using the Short Sensory Profile caregiver questionnaire (McIntosh et al., 1999; Licciardi and Brown, 2021). A score of ≥2 standard deviations from the mean in any of the following domains corresponds to a SPD designation: tactile sensitivity, taste/smell sensitivity, movement sensitivity, under-responsive/seeks sensation, auditory filtering, low energy/weak, visual/auditory sensitivity. ADHD was assessed with the Behavior Assessment for Children: Third Edition (BASC-3) with a categorization of clinical significance, using a 95th percentile threshold, corresponding to an ADHD label (Reynolds and Kamphaus, 2015).

### MR imaging, DTI and NODDI acquisition

All subjects were imaged on a single Siemens 3 Tesla (3 T) Prisma MRI scanner (Erlangen, Germany) using a 64-channel head coil. Structural MRI of the brain was acquired with an axial 3D magnetization prepared rapid acquisition gradient-echo (MPRAGE) T1-weighted sequence. Whole brain diffusion MRI was performed at diffusion-weighting strengths (shells) of b = 1,000 s/mm^2^ (64 diffusion-encoding directions, 5 b = 0 s/mm^2^) and 2,500 s/mm^2^ (96 diffusion-encoding directions, 10 b = 0 s/mm^2^) (TE = 72.20 ms, TR = 2,420 ms, flip angle = 85 degrees, slice thickness = 2.0 mm, matrix size = 110×110, FOV = 220 mm) using single-shot spin echo echoplanar imaging with additional paired forward and reverse phase encoding b = 0 s/mm^2^ volumes. Simultaneous multiband (MB) excitation was used (MB factor = 3). The acquisition time for the b = 1,000 s/mm^2^ shell was 3 min and 23 s, and for the b = 2,500 s/mm^2^ shell was 4 min and 53 s.

### Diffusion MR image processing and analysis

Each participant’s dMRI data underwent quality control inspections and the same processing pipeline to compute DTI and NODDI metrics. First, the data were denoised using the Marcenko-Pastur Principal Component Analysis algorithm [MP-PCA; (Veraart et al., 2016)] through the Dipy library (Garyfallidis et al., 2014). The FMRIB Software Library (FSL) version 6.0.2 (Oxford, United Kingdpm) was used for diffusion processing and DTI parameter computation per steps previously reported (Owen et al., 2013; Chang et al., 2015a,b; Payabvash et al., 2019). FSL’s topup was ran using the participant’s paired forward and reversed phase encoding images and applied on each diffusion shell to correct for susceptibility induced distortion (Andersson et al., 2003). The b = 1,000 s/mm^2^ and b = 2,500 s/mm^2^ scans were then concatenated. A brain mask was created from the first volume of the multi-shell data using Freesurfer’s SynthStrip (Hoopes et al., 2022). FSL’s Eddy was used on the diffusion data to correct for motion and eddy distortions, skull stripping, outlier replacement, susceptibility-by-movement, and slice-to-volume correction. The b = 1,000 s/mm^2^ shell was extracted from the processed multi-shell data and used to calculate DTI parameters. To determine the signal-to-noise ratio of the dMRI acquisition, the the ratio of the mean of the b = 0 s/mm^2^ signal was divided by the standard deviation of the underlying Gaussian noise, using the method described in https://dipy.org/documentation/1.0.0./examples_built/snr_in_cc/. To increase SNR, the b = 0 s/mm^2^ volumes were averaged together and used as the first volume followed by remaining 64 diffusion-weighted volumes; this input was used in FSL’s dtifit to calculate FA, MA, RD and AD maps. The processed multi-shell data was used to quantify NODDI parameters: NDI, ODI, and FISO with the Accelerated Microstructure Imaging via Convex Optimization (AMICO) Toolbox (Daducci et al., 2015). Example FA, directionally-encoded color FA, AD, NDI and ODI maps are shown in Supplementary Figure S1.

TBSS in FSL (Smith et al., 2006) was used to skeletonize and register the diffusion metric maps of each participant in order to perform global and region of interest (ROI) measurements along the white matter skeleton. Using TBSS, “the most representative subject” was determined from the FA maps of all participants and used as the target image, as recommended for populations of young children. The target image was affine-aligned into MNI152 standard space. Each FA map was transformed by combining the non-linear transform to the target FA image and the affine transform from the determined target image to MNI152 space and resampled to 1 mm resolution. The registered FA maps were then averaged and thinned to generate a mean FA skeleton to represent the core of all white matter tracts. The FA white matter skeleton was thresholded to FA > 0.2 to exclude voxels containing gray matter and partial volume effects. Each participant’s MD, AD, RD, NDI, ODI, and FISO maps were then registered and projected onto the white matter skeleton to create skeletonized maps of each diffusion metric. Global WM analysis was performed by averaging each diffusion metric from each participant within the skeletonized white matter mask calculated from the standardized FA TBSS results. The Johns Hopkins University (JHU) ICBM-DTI-81 White-Matter Labeled Atlas was used to extract the average DTI and NODDI values. Forty-three white matter tracts were included in the analysis; FX, FXST and TPT regions were excluded due to their small size and unreliability (Table 1).

**Table 1:** This provides a list and abbreviations of the JHU atlas white matter tracts used in the TBSS analysis.

White matter tract abbreviations
*Commissural tracts* (corticocortical tracts connecting left and right hemispheres): splenium (SCC), body (BCC), and genu (GCC) of the corpus callosum;
*Brainstem tracts*: inferior (ICP), middle (MCP), and superior (SCP) cerebellar peduncles, pontine crossing tract (PCT), and medial lemniscus (ML);
*Projection tracts* (cortical to subcortical regions): corticospinal tract (CST), cerebral peduncle (CP), posterior thalamic radiation (PTR), internal capsule (subdivided into anterior limb (ALIC), posterior limb (PLIC), and retrolenticular (RLIC) portions), and corona radiata (subdivided into anterior (ACR), superior (SCR), and posterior (PCR) portions);
*Limbic tracts*: cingulum (subdivided into cingulate (CGC) and hippocampal (CGH) portions), fornix (FX), and fornix stria terminalis (FXST)
*Association tracts* (corticocortical tracts within same hemisphere): external capsule (EC), superior fronto-occipital fasciculus (SFO), superior longitudinal fasciculus (SLF), sagittal stratum (SS), and uncinate fasciculus (UNC).
*Laterality:* Denoted by the tract followed by “-L” for left or “-R” for right.

### Statistics

Unpaired two-tailed *t*-tests were run to determine group differences between SPD+ADHD and SPD-ADHD cohorts with DTI and NODDI metrics using a statistical significance threshold of 𝛼=0.05. A False Discovery Rate [FDR; (Benjamini and Hochberg, 1995)] adjustment was made on the *p*-values to correct for multiple comparisons for the JHU white matter regions within each metric. T-statistics and other descriptive statistics including mean, standard deviation and Cohen’s D effect size were performed with Python v3.7.6 (Python Software Foundation, https://www.python.org/) statistical packages. A data-driven voxelwise analysis was performed on diffusion metrics to further explore group comparisons that resulted in significant differences in white matter ROIs. This was done with a general linear model with permutation testing implemented with FSL’s randomise (Winkler et al., 2014) and corrected for multiple voxelwise comparisons using threshold-free cluster enhancement [TFCE; (Smith and Nichols, 2009)] and family-wise error (FWE) corrected at *p* < 0.05.

## Results

### Demographics

One hundred subjects with SPD were included in the study, with age, sex and intelligence quotient (IQ) scores given in Table 2.

**Table 2:** Age, sex distribution and WISC-V IQ scores of the SPD + ADHD and SPD-ADHD cohorts.

Group	*n*	Age (μ ± 𝜎)	WISC V					
			FSIQ (μ ± 𝜎)	FRI (μ ± 𝜎)	PSI (μ ± 𝜎)	VCI (μ ± 𝜎)	VSI (μ ± 𝜎)	WMI (μ ± 𝜎)
SPD+ADHD	36	9.99 ± 1.58	102.69 ± 14.50	105.11 ± 14.25	90.50 ± 13.37	109.22 ± 13.73	106.44 ± 12.16	99.53 ± 14.56
Male SPD+ADHD	28	10.01 ± 1.50	102.64 ± 14.54	105.32 ± 14.84	88.71 ± 12.01	110.04 ± 14.40	107.71 ± 12.28	99.57 ± 14.55
Female SPD+ADHD	8	9.92 ± 1.97	102.88 ± 15.37	104.38 ± 12.82	96.75 ± 16.75	106.38 ± 11.44	102.00 ± 11.38	99.38 ± 15.60
SPD-ADHD	64	10.12 ± 1.57	104.81 ± 13.16	107.11 ± 13.31	92.91 ± 12.95	109.73 ± 14.70	107.67 ± 13.44	100.73 ± 15.59
Male SPD-ADHD	38	10.06 ± 1.54	104.53 ± 11.64	107.61 ± 13.01	90.37 ± 11.39	109.37 ± 14.42	108.03 ± 12.24	101.24 ± 15.24
Female SPD-ADHD	26	10.21 ± 1.64	105.23 ± 15.35	106.38 ± 13.97	96.62 ± 14.38	110.27 ± 15.37	107.15 ± 15.28	100.00 ± 16.37

FSIQ, Full-Scale Intelligence Quotient; FRI, Fluid Reasoning Index; PSI, Processing Speed Index; VCI, Verbal Comprehension Index; VSI, Visuospatial Index; WMI, Working Memory Index.

### Global white matter analysis

**Figure 1:**
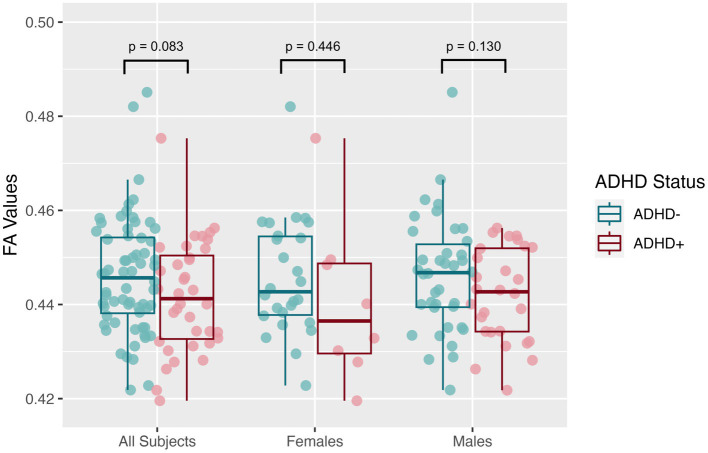
Global white matter fractional anisotropy (FA) values from diffusion tensor imaging (DTI) for the SPD + ADHD group compared to the SPD-ADHD group, as well as for males only and for females only.

**Figure 2:**
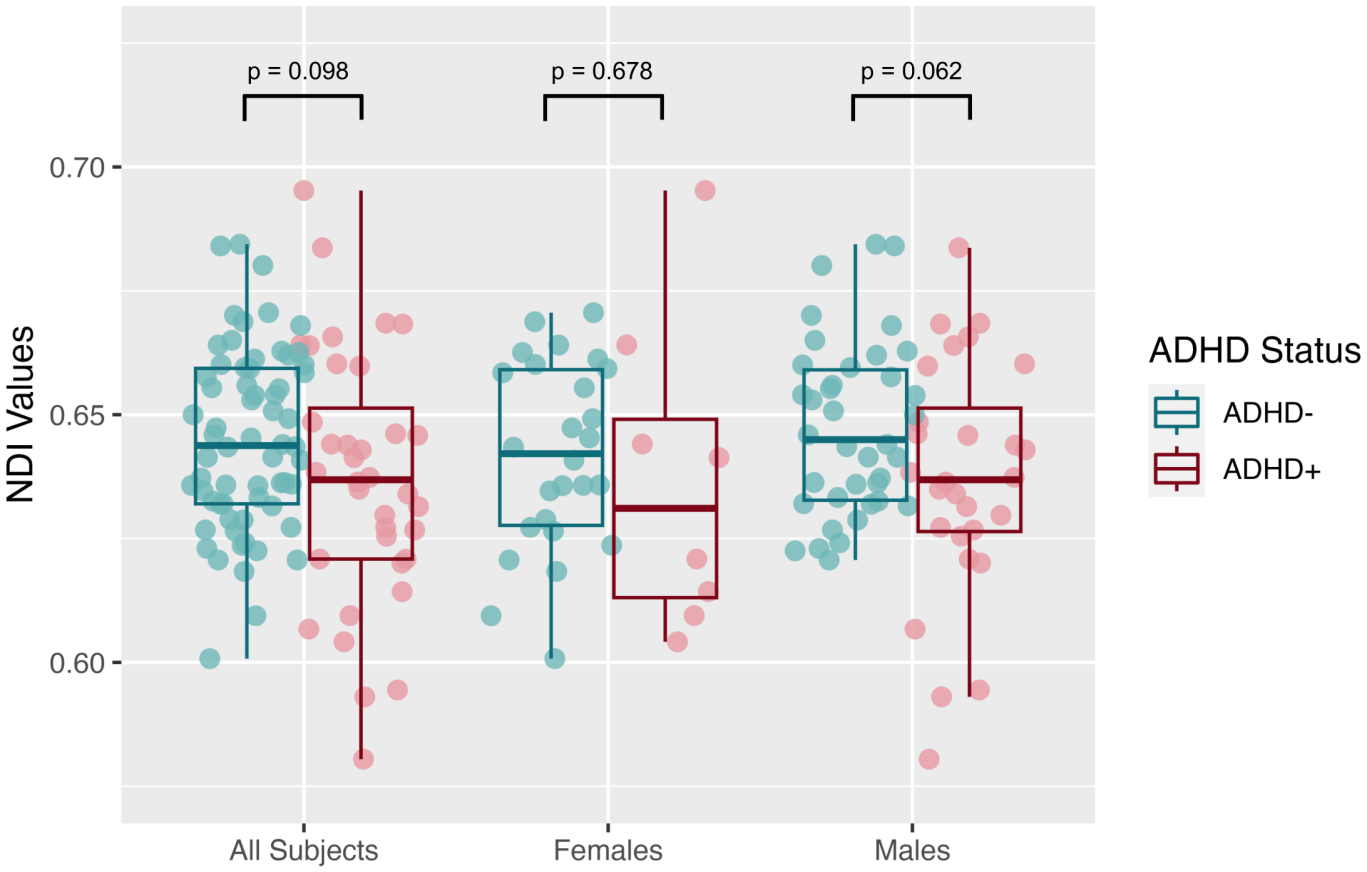
Global white matter neurite density index (NDI) values from neurite orientation density and dispersion imaging (NODDI) for the SPD + ADHD group compared to the SPD-ADHD group, as well as for males only and for females only.

The mean SNR across single b = 0 s/mm^2^ volumes from the participants was 49.4 ± 7.4, more than twice the threshold of 20 traditionally recommended for DTI. DTI assessing the global white matter revealed FA in the SPD+ADHD group trended lower than in the SPD-ADHD group (*p* = 0.083) with a small effect size (Cohen’s D = −0.36), directionally consistent with our hypothesis, that did not differ between boys and girls (Figure 1). The NODDI analysis (Figure 2) also revealed a trend towards lower global WM NDI in the SPD+ADHD group (*p* = 0.098, Cohen’s D = −0.39), congruent with our hypothesis. This effect was larger in males reaching medium effect size (*p* = 0.062, Cohen’s D = −0.50), than in females (*p* = 0.68, Cohen’s D = −0.22). Post-hoc exploratory analyses of other NODDI metrics showed global WM FISO trended lower in boys with SPD+ADHD versus boys with SPD-ADHD (*p* = 0.057, Cohen’s D = −0.50).

### Voxelwise white matter analysis

**Figure 3:**
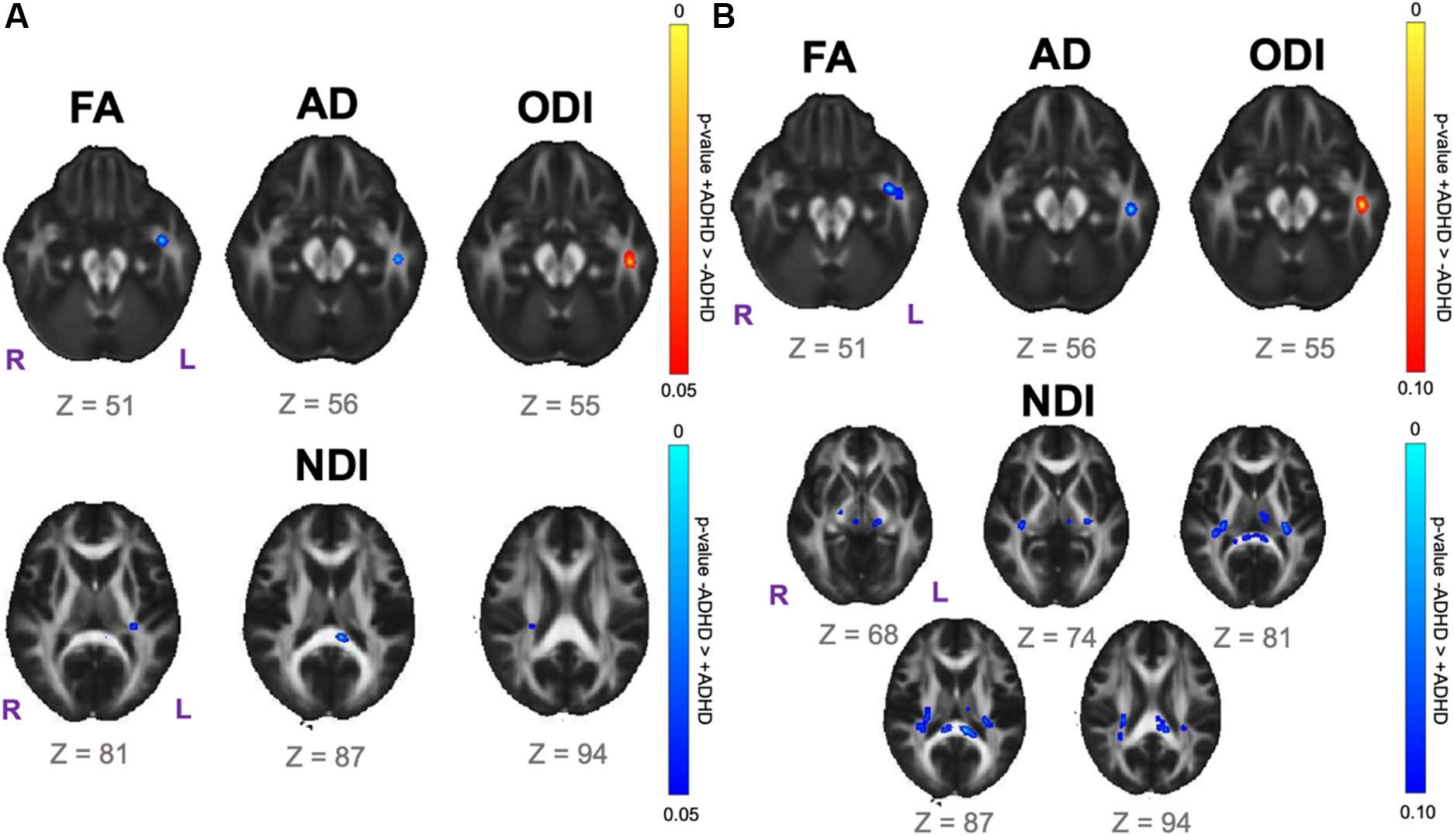
Voxelwise white matter group comparison maps from tract-based spatial statistics (TBSS) for the SPD + ADHD group compared to the SPD-ADHD group for boys only. FA: fractional anisotropy. AD: axial diffusivity. ODI: orientation dispersion index. NDI: neurite density index. **(A)** Statistically significant voxel clusters at *p* < 0.05 corrected for multiple voxelwise comparisons. **(B)** Trend-level voxel clusters at *p* < 0.10 corrected for multiple voxelwise comparisons.

Data-driven voxelwise DTI and NODDI analyses using TBSS did not reveal statistically significant results for the SPD+ADHD vs. SPD-ADHD group comparison after correction for multiple voxelwise comparisons. However, investigation of boys only reveals a focus of decreased FA in the left uncinate fasciculus (UNC-L) for the SPD+ADHD group compared to the SPD-ADHD group at *p* < 0.05 (corrected; Figure 3A). There is also a focus of decreased FA in the left inferior longitudinal fasciculus (ILF-L) that corresponds to increased ODI on NODDI. Decreased NDI is observed for boys with SPD+ADHD versus SPD-ADHD in the SCC, PCR-R and RLIC-L (see Table 1 for tract abbreviations). Examining for trend-level group differences in boys at *p* < 0.10 (corrected) shows expanded foci of low NDI in the SCC, bilateral RLIC, PCR-R, PLIC-R and CP-R (Figure 3B).

### Regional tract-specific analysis

**Figure 4:**
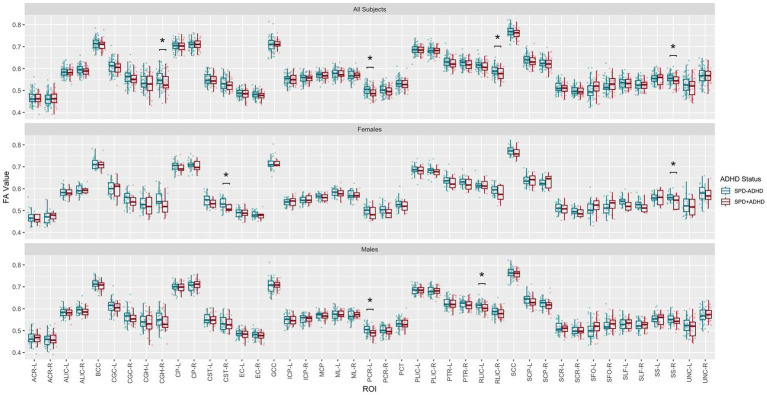
Johns Hopkins University White Matter Atlas tract-level fractional anisotropy (FA) values from diffusion tensor imaging (DTI) for the SPD + ADHD group compared to the SPD-ADHD group, as well as for males only and for females only. Tract abbreviations are provided in Table 1. A single asterisk denotes a group difference of p < 0.05 that is uncorrected for multiple comparisons and a double asterisk denotes a group difference of p < 0.05 that is FDR-corrected for multiple comparisons.

**Figure 5:**
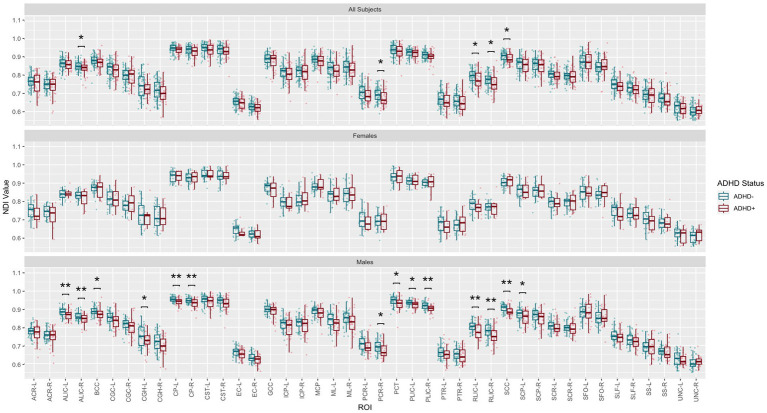
Johns Hopkins University White Matter Atlas tract-level neurite density index (NDI) values from neurite orientation density and dispersion imaging (NODDI) for the SPD + ADHD group compared to the SPD-ADHD group, as well as for males only and for females only. Tract abbreviations are provided in Table 1. A single asterisk denotes a group difference of *p* < 0.05 that is uncorrected for multiple comparisons and a double asterisk denotes a group difference of *p* < 0.05 that is FDR-corrected for multiple comparisons.

Post-hoc exploratory analysis of the JHU Atlas based white matter tracts (see tract abbreviations in Table 1) was performed to further localize the white matter microstructural differences between groups. Analysis of all children showed 5 tracts (CGH-R, SS-R, PCR-L, and RLIC-R) that had lower FA in the SPD+ADHD group at p < 0.05 (Figure 4 and Table 3), but these did not survive correction for multiple comparisons. Relative to FA, there were more robust group differences for NDI (Figure 5 and Table 4). Although none of the NDI group differences for all children survived multiple comparisons correction, when evaluating only boys, eight tracts had significantly lower NDI in SPD+ADHD than in SPD-ADHD (ALIC-L, ALIC-R, CP-L, CP-R, PLIC-R, RLIC-L, RLIC-R and SCC) after multiple comparisons correction. These tract-level results correspond well to the data-driven voxelwise results for NDI in boys only (Figure 3).

**Table 3:** Differences of FA regional values between SPD + ADHD and SPD-ADHD groups.

Region	*All*				*Female*				*Male*			
	*μ*_SPD + ADHD_ *± σ*	*μ*_SPD-ADHD_ *± σ*	*d*	*p*	*μ*_SPD + ADHD_ *± σ*	*μ*_SPD-ADHD_ *± σ*	*d*	*p*	*μ*_SPD + ADHD_ *± σ*	*μ*_SPD-ADHD_ *± σ*	*d*	*p*
CGC-L	0.602 ± 0.027	0.609 ± 0.032	−0.244	0.255	0.599 ± 0.045	0.603 ± 0.033	−0.096	0.794	0.604 ± 0.022	0.614 ± 0.032	−0.368	0.156
CGC-R	0.554 ± 0.030	0.560 ± 0.032	−0.201	0.344	0.543 ± 0.039	0.554 ± 0.032	−0.299	0.437	0.558 ± 0.027	0.566 ± 0.032	−0.262	0.304
CGH-L	0.530 ± 0.043	0.539 ± 0.033	−0.257	0.203	0.520 ± 0.046	0.537 ± 0.036	−0.416	0.276	0.534 ± 0.043	0.543 ± 0.031	−0.243	0.322
CGH-R	0.535 ± 0.042	0.554 ± 0.037	**−0.465**	0.025*	0.523 ± 0.048	0.553 ± 0.037	−0.687	0.076	0.538 ± 0.041	0.553 ± 0.038	−0.380	0.13
EC-L	0.485 ± 0.022	0.488 ± 0.017	−0.149	0.460	0.488 ± 0.026	0.490 ± 0.019	−0.053	0.886	0.484 ± 0.021	0.487 ± 0.016	−0.166	0.498
EC-R	0.478 ± 0.020	0.483 ± 0.016	−0.320	0.118	0.478 ± 0.020	0.483 ± 0.014	−0.304	0.411	0.477 ± 0.020	0.482 ± 0.018	−0.299	0.229
SFO-L	0.515 ± 0.037	0.502 ± 0.038	0.336	0.112	0.517 ± 0.039	0.508 ± 0.037	0.246	0.543	0.517 ± 0.035	0.504 ± 0.039	0.343	0.176
SFO-R	0.530 ± 0.032	0.519 ± 0.029	0.350	0.092	0.527 ± 0.041	0.510 ± 0.028	0.486	0.188	0.531 ± 0.028	0.525 ± 0.028	0.207	0.41
SLF-L	0.532 ± 0.025	0.536 ± 0.024	−0.170	0.413	0.525 ± 0.029	0.543 ± 0.022	−0.721	0.061	0.532 ± 0.023	0.531 ± 0.025	0.038	0.88
SLF-R	0.525 ± 0.022	0.528 ± 0.023	−0.165	0.434	0.515 ± 0.027	0.530 ± 0.021	−0.638	0.099	0.526 ± 0.020	0.525 ± 0.024	0.023	0.927
SS-L	0.552 ± 0.028	0.557 ± 0.021	−0.168	0.404	0.560 ± 0.037	0.560 ± 0.018	0.011	0.973	0.552 ± 0.026	0.556 ± 0.023	−0.175	0.482
SS-R	0.544 ± 0.028	0.557 ± 0.020	**−0.513**	0.011*	0.541 ± 0.033	0.562 ± 0.018	**−0.786**	0.025*	0.545 ± 0.026	0.552 ± 0.021	−0.300	0.225
UNC-L	0.518 ± 0.041	0.528 ± 0.037	−0.251	0.225	0.519 ± 0.043	0.528 ± 0.043	−0.220	0.592	0.516 ± 0.042	0.523 ± 0.032	−0.188	0.444
UNC-R	0.568 ± 0.034	0.568 ± 0.034	0.004	0.984	0.571 ± 0.042	0.577 ± 0.037	−0.161	0.684	0.575 ± 0.032	0.570 ± 0.034	0.132	0.599
ACR-L	0.463 ± 0.025	0.468 ± 0.029	−0.200	0.349	0.461 ± 0.020	0.469 ± 0.031	−0.305	0.500	0.464 ± 0.026	0.468 ± 0.028	−0.143	0.569
ACR-R	0.465 ± 0.027	0.464 ± 0.028	0.042	0.842	0.475 ± 0.019	0.468 ± 0.028	0.289	0.520	0.461 ± 0.029	0.460 ± 0.027	0.041	0.871
PCR-L	0.490 ± 0.024	0.504 ± 0.022	**−0.628**	0.003*	0.489 ± 0.033	0.501 ± 0.023	−0.440	0.233	0.489 ± 0.022	0.505 ± 0.022	**−0.733**	0.004*
PCR-R	0.496 ± 0.020	0.503 ± 0.023	−0.362	0.090	0.491 ± 0.026	0.504 ± 0.023	−0.527	0.184	0.498 ± 0.019	0.504 ± 0.023	−0.291	0.255
SCR-L	0.509 ± 0.022	0.515 ± 0.024	−0.245	0.248	0.508 ± 0.032	0.513 ± 0.023	−0.177	0.635	0.508 ± 0.019	0.514 ± 0.025	−0.266	0.299
SCR-R	0.497 ± 0.019	0.500 ± 0.018	−0.187	0.370	0.490 ± 0.022	0.497 ± 0.018	−0.343	0.374	0.499 ± 0.019	0.502 ± 0.019	−0.138	0.581
ALIC-L	0.581 ± 0.019	0.584 ± 0.020	−0.140	0.507	0.581 ± 0.024	0.584 ± 0.023	−0.100	0.802	0.581 ± 0.018	0.583 ± 0.019	−0.130	0.603
ALIC-R	0.590 ± 0.018	0.595 ± 0.020	−0.273	0.201	0.595 ± 0.018	0.594 ± 0.022	0.024	0.956	0.589 ± 0.019	0.597 ± 0.020	−0.395	0.119
PLIC-L	0.683 ± 0.018	0.686 ± 0.021	−0.134	0.529	0.680 ± 0.024	0.687 ± 0.024	−0.288	0.479	0.685 ± 0.017	0.686 ± 0.019	−0.033	0.896
PLIC-R	0.681 ± 0.019	0.683 ± 0.021	−0.082	0.699	0.676 ± 0.019	0.684 ± 0.023	−0.362	0.397	0.683 ± 0.018	0.682 ± 0.021	0.046	0.856
RLIC-L	0.607 ± 0.024	0.615 ± 0.021	−0.342	0.097	0.617 ± 0.028	0.614 ± 0.022	0.136	0.718	0.604 ± 0.023	0.616 ± 0.021	**−0.51**	0.043*
RLIC-R	0.577 ± 0.027	0.591 ± 0.023	**−0.548**	0.008*	0.577 ± 0.038	0.595 ± 0.023	−0.569	0.113	0.578 ± 0.023	0.589 ± 0.022	−0.479	0.058
CST-L	0.547 ± 0.024	0.553 ± 0.025	−0.258	0.222	0.533 ± 0.021	0.550 ± 0.029	−0.661	0.140	0.550 ± 0.024	0.554 ± 0.022	−0.176	0.48
CST-R	0.526 ± 0.029	0.532 ± 0.028	−0.207	0.323	0.511 ± 0.015	0.531 ± 0.025	**−0.961**	0.042*	0.532 ± 0.031	0.533 ± 0.029	−0.055	0.825
PTR-L	0.624 ± 0.023	0.632 ± 0.025	−0.303	0.153	0.626 ± 0.027	0.639 ± 0.026	−0.517	0.207	0.624 ± 0.023	0.626 ± 0.023	−0.083	0.741
PTR-R	0.618 ± 0.024	0.626 ± 0.021	−0.359	0.081	0.619 ± 0.030	0.632 ± 0.020	−0.515	0.163	0.618 ± 0.023	0.622 ± 0.020	−0.188	0.449
BCC	0.708 ± 0.023	0.714 ± 0.022	−0.290	0.163	0.712 ± 0.031	0.714 ± 0.024	−0.067	0.859	0.706 ± 0.021	0.714 ± 0.021	−0.379	0.133
GCC	0.711 ± 0.021	0.710 ± 0.032	0.020	0.928	0.718 ± 0.022	0.714 ± 0.029	0.162	0.713	0.708 ± 0.020	0.707 ± 0.033	0.037	0.887
SCC	0.760 ± 0.021	0.767 ± 0.023	−0.317	0.134	0.763 ± 0.027	0.772 ± 0.021	−0.401	0.294	0.760 ± 0.020	0.765 ± 0.023	−0.196	0.439
CP-L	0.702 ± 0.022	0.703 ± 0.021	−0.015	0.941	0.697 ± 0.029	0.704 ± 0.022	−0.272	0.466	0.701 ± 0.020	0.699 ± 0.020	0.096	0.701
CP-R	0.709 ± 0.023	0.708 ± 0.022	0.056	0.789	0.702 ± 0.028	0.709 ± 0.023	−0.299	0.444	0.711 ± 0.022	0.706 ± 0.021	0.264	0.292
ICP-L	0.551 ± 0.022	0.551 ± 0.023	−0.022	0.917	0.541 ± 0.025	0.537 ± 0.018	0.157	0.668	0.549 ± 0.020	0.551 ± 0.025	−0.067	0.791
ICP-R	0.555 ± 0.018	0.557 ± 0.021	−0.074	0.728	0.550 ± 0.021	0.548 ± 0.018	0.131	0.733	0.553 ± 0.017	0.555 ± 0.021	−0.068	0.789
MCP	0.568 ± 0.017	0.570 ± 0.015	−0.095	0.644	0.559 ± 0.019	0.565 ± 0.013	−0.360	0.333	0.569 ± 0.016	0.569 ± 0.016	−0.031	0.9
SCP-L	0.633 ± 0.027	0.640 ± 0.022	−0.308	0.133	0.637 ± 0.030	0.637 ± 0.024	−0.035	0.928	0.632 ± 0.027	0.643 ± 0.021	−0.456	0.066
SCP-R	0.621 ± 0.029	0.625 ± 0.020	−0.166	0.401	0.633 ± 0.033	0.625 ± 0.021	0.293	0.412	0.617 ± 0.028	0.626 ± 0.020	−0.367	0.134
ML-L	0.577 ± 0.019	0.578 ± 0.022	−0.027	0.898	0.580 ± 0.028	0.584 ± 0.018	−0.140	0.698	0.577 ± 0.016	0.576 ± 0.024	0.061	0.813
ML-R	0.571 ± 0.017	0.569 ± 0.021	0.125	0.559	0.572 ± 0.030	0.572 ± 0.020	0.017	0.961	0.574 ± 0.013	0.569 ± 0.022	0.237	0.362
PCT	0.530 ± 0.024	0.532 ± 0.024	−0.119	0.568	0.519 ± 0.026	0.531 ± 0.026	−0.467	0.258	0.533 ± 0.024	0.533 ± 0.023	−0.002	0.994

For *t*-test, BOLD Cohen’s d is for *p* < 0.05 (uncorrected), a single asterisk is for *p* < 0.05 (uncorrected) and two asterisks is for FDR-adjusted *p* < 0.05. Tract abbreviations are as in Table 1.

**Table 4:** Differences of NDI regional values between SPD + ADHD and SPD-ADHD groups.

Region	*All*				*Female*				*Male*			
	*μ*_SPD + ADHD_ *± σ*	*μ*_SPD-ADHD_ *± σ*	*d*	*p*	*μ*_SPD + ADHD_ *± σ*	*μ*_SPD-ADHD_ *± σ*	*d*	*p*	*μ*_SPD + ADHD_ *± σ*	*μ*_SPD-ADHD_ *± σ*	*d*	*p*
CGC-L	0.830 ± 0.051	0.836 ± 0.042	−0.131	0.519	0.817 ± 0.064	0.813 ± 0.041	0.070	0.844	0.836 ± 0.046	0.854 ± 0.033	−0.454	0.066
CGC-R	0.797 ± 0.055	0.796 ± 0.039	0.007	0.971	0.789 ± 0.062	0.774 ± 0.035	0.300	0.385	0.799 ± 0.054	0.813 ± 0.035	−0.307	0.206
CGH-L	0.723 ± 0.054	0.743 ± 0.064	−0.340	0.114	0.721 ± 0.065	0.730 ± 0.071	−0.132	0.752	0.724 ± 0.052	0.753 ± 0.058	**−0.522**	**0.042** ^*^
CGH-R	0.701 ± 0.055	0.720 ± 0.057	−0.334	0.114	0.717 ± 0.065	0.718 ± 0.063	−0.021	0.959	0.697 ± 0.051	0.721 ± 0.053	−0.473	0.062
EC-L	0.647 ± 0.031	0.657 ± 0.029	−0.319	0.127	0.634 ± 0.035	0.646 ± 0.030	−0.383	0.330	0.652 ± 0.029	0.665 ± 0.026	−0.477	0.058
EC-R	0.620 ± 0.029	0.628 ± 0.024	−0.301	0.140	0.618 ± 0.035	0.622 ± 0.021	−0.144	0.682	0.621 ± 0.029	0.633 ± 0.025	−0.427	0.088
SFO-L	0.874 ± 0.055	0.871 ± 0.047	0.069	0.735	0.853 ± 0.054	0.854 ± 0.049	−0.027	0.946	0.881 ± 0.054	0.883 ± 0.041	−0.046	0.851
SFO-R	0.853 ± 0.055	0.848 ± 0.042	0.118	0.555	0.837 ± 0.064	0.839 ± 0.037	−0.045	0.896	0.859 ± 0.051	0.854 ± 0.043	0.118	0.633
SLF-L	0.743 ± 0.042	0.753 ± 0.036	−0.260	0.206	0.733 ± 0.062	0.753 ± 0.038	−0.374	0.293	0.747 ± 0.036	0.754 ± 0.036	−0.213	0.396
SLF-R	0.723 ± 0.041	0.734 ± 0.034	−0.278	0.173	0.734 ± 0.051	0.734 ± 0.032	0.008	0.982	0.720 ± 0.038	0.733 ± 0.035	−0.368	0.142
SS-L	0.689 ± 0.051	0.697 ± 0.042	−0.167	0.410	0.687 ± 0.047	0.704 ± 0.050	−0.340	0.416	0.691 ± 0.053	0.694 ± 0.034	−0.053	0.826
SS-R	0.665 ± 0.053	0.675 ± 0.037	−0.222	0.264	0.693 ± 0.058	0.682 ± 0.036	0.224	0.528	0.657 ± 0.050	0.670 ± 0.037	−0.299	0.224
UNC-L	0.612 ± 0.037	0.626 ± 0.041	−0.355	0.096	0.614 ± 0.044	0.624 ± 0.038	−0.232	0.552	0.613 ± 0.036	0.629 ± 0.045	−0.376	0.142
UNC-R	0.601 ± 0.035	0.600 ± 0.035	0.028	0.893	0.621 ± 0.038	0.607 ± 0.033	0.393	0.319	0.602 ± 0.034	0.602 ± 0.038	−0.011	0.965
ACR-L	0.759 ± 0.053	0.767 ± 0.040	−0.175	0.382	0.735 ± 0.062	0.751 ± 0.043	−0.290	0.429	0.766 ± 0.050	0.778 ± 0.034	−0.285	0.243
ACR-R	0.740 ± 0.058	0.752 ± 0.033	−0.261	0.178	0.721 ± 0.069	0.743 ± 0.034	−0.413	0.218	0.745 ± 0.054	0.758 ± 0.031	−0.293	0.224
PCR-L	0.693 ± 0.046	0.707 ± 0.043	−0.311	0.136	0.689 ± 0.051	0.702 ± 0.052	−0.239	0.562	0.694 ± 0.045	0.711 ± 0.037	−0.401	0.106
PCR-R	0.674 ± 0.045	0.693 ± 0.039	**−0.436**	0.035*	0.693 ± 0.054	0.692 ± 0.043	0.029	0.939	0.669 ± 0.041	0.694 ± 0.037	**−0.632**	**0.013** ^*^
SCR-L	0.804 ± 0.042	0.806 ± 0.036	−0.044	0.828	0.798 ± 0.054	0.796 ± 0.038	0.045	0.904	0.804 ± 0.039	0.810 ± 0.034	−0.174	0.483
SCR-R	0.795 ± 0.043	0.799 ± 0.033	−0.108	0.593	0.797 ± 0.051	0.793 ± 0.032	0.108	0.761	0.794 ± 0.041	0.803 ± 0.034	−0.241	0.329
ALIC-L	0.861 ± 0.029	0.866 ± 0.033	−0.152	0.475	0.843 ± 0.030	0.838 ± 0.028	0.159	0.689	0.866 ± 0.028	0.884 ± 0.024	**−0.696**	**0.006** ^**^
ALIC-R	0.837 ± 0.036	0.850 ± 0.026	**−0.419**	0.037*	0.815 ± 0.052	0.834 ± 0.023	−0.481	0.144	0.843 ± 0.029	0.861 ± 0.021	**−0.700**	**0.005** ^**^
PLIC-L	0.921 ± 0.025	0.925 ± 0.023	−0.157	0.449	0.914 ± 0.033	0.910 ± 0.025	0.139	0.709	0.923 ± 0.022	0.935 ± 0.016	**−0.618**	**0.013** ^*^
PLIC-R	0.902 ± 0.032	0.912 ± 0.021	−0.351	0.077	0.898 ± 0.052	0.902 ± 0.017	−0.114	0.704	0.903 ± 0.024	0.919 ± 0.020	**−0.700**	**0.006** ^**^
RLIC-L	0.775 ± 0.047	0.797 ± 0.037	**−0.518**	0.012*	0.772 ± 0.052	0.784 ± 0.044	−0.254	0.513	0.776 ± 0.046	0.806 ± 0.031	**−0.756**	**0.003** ^**^
RLIC-R	0.755 ± 0.051	0.776 ± 0.037	**−0.468**	0.021*	0.770 ± 0.055	0.767 ± 0.035	0.065	0.855	0.751 ± 0.050	0.782 ± 0.038	**−0.677**	**0.007** ^**^
CST-L	0.942 ± 0.034	0.949 ± 0.029	−0.231	0.260	0.950 ± 0.027	0.946 ± 0.030	0.124	0.768	0.940 ± 0.036	0.952 ± 0.029	−0.369	0.136
CST-R	0.932 ± 0.039	0.941 ± 0.032	−0.253	0.214	0.941 ± 0.031	0.938 ± 0.034	0.089	0.830	0.929 ± 0.041	0.944 ± 0.029	−0.408	0.098
PTR-L	0.654 ± 0.049	0.671 ± 0.043	−0.368	0.075	0.663 ± 0.051	0.683 ± 0.051	−0.393	0.338	0.651 ± 0.049	0.663 ± 0.035	−0.261	0.286
PTR-R	0.646 ± 0.052	0.660 ± 0.039	−0.299	0.139	0.682 ± 0.055	0.673 ± 0.042	0.183	0.627	0.636 ± 0.047	0.651 ± 0.035	−0.358	0.147
BCC	0.871 ± 0.039	0.881 ± 0.030	−0.291	0.150	0.868 ± 0.052	0.870 ± 0.036	−0.037	0.918	0.872 ± 0.035	0.888 ± 0.023	**−0.561**	**0.023** ^*^
GCC	0.880 ± 0.046	0.891 ± 0.031	−0.279	0.159	0.862 ± 0.058	0.878 ± 0.033	−0.328	0.341	0.885 ± 0.041	0.900 ± 0.026	−0.430	0.079
SCC	0.893 ± 0.031	0.906 ± 0.025	**−0.455**	0.027*	0.909 ± 0.038	0.904 ± 0.029	0.135	0.721	0.889 ± 0.028	0.908 ± 0.022	**−0.740**	**0.004** ^**^
CP-L	0.941 ± 0.022	0.948 ± 0.025	−0.302	0.156	0.937 ± 0.031	0.936 ± 0.031	0.010	0.980	0.941 ± 0.020	0.956 ± 0.016	**−0.794**	**0.002** ^**^
CP-R	0.930 ± 0.029	0.941 ± 0.024	−0.393	0.055	0.929 ± 0.038	0.930 ± 0.029	−0.025	0.946	0.931 ± 0.027	0.948 ± 0.017	**−0.771**	**0.002** ^**^
ICP-L	0.805 ± 0.056	0.820 ± 0.040	−0.294	0.143	0.798 ± 0.057	0.800 ± 0.044	−0.037	0.922	0.802 ± 0.056	0.820 ± 0.035	−0.380	0.118
ICP-R	0.815 ± 0.060	0.822 ± 0.040	−0.155	0.434	0.818 ± 0.059	0.808 ± 0.042	0.195	0.597	0.810 ± 0.060	0.822 ± 0.039	−0.247	0.31
MCP	0.879 ± 0.038	0.887 ± 0.028	−0.245	0.222	0.886 ± 0.042	0.882 ± 0.029	0.127	0.729	0.875 ± 0.037	0.887 ± 0.028	−0.383	0.12
SCP-L	0.854 ± 0.040	0.866 ± 0.037	−0.321	0.122	0.859 ± 0.047	0.861 ± 0.041	−0.027	0.946	0.852 ± 0.039	0.871 ± 0.034	**−0.505**	**0.045** ^*^
SCP-R	0.852 ± 0.046	0.859 ± 0.040	−0.179	0.384	0.862 ± 0.046	0.855 ± 0.045	0.158	0.697	0.849 ± 0.046	0.863 ± 0.035	−0.361	0.143
ML-L	0.826 ± 0.053	0.840 ± 0.047	−0.278	0.179	0.841 ± 0.049	0.838 ± 0.053	0.057	0.892	0.821 ± 0.054	0.841 ± 0.043	−0.400	0.106
ML-R	0.832 ± 0.059	0.847 ± 0.047	−0.278	0.172	0.842 ± 0.058	0.844 ± 0.055	−0.035	0.931	0.830 ± 0.059	0.850 ± 0.042	−0.384	0.118
PCT	0.930 ± 0.040	0.940 ± 0.032	−0.297	0.143	0.934 ± 0.045	0.933 ± 0.036	0.013	0.972	0.929 ± 0.039	0.946 ± 0.028	**−0.502**	**0.043** ^*^

For *t*-test, BOLD Cohen’s d is for *p* < 0.05 (uncorrected), a single asterisk is for *p* < 0.05 (uncorrected) and two asterisks is for FDR-adjusted *p* < 0.05. Tract abbreviations are as in Table 1.

## Discussion

Our study is the first to evaluate the neuroarchitecture of the attentional dimension of SPD, and as such should shed light on the neurobiological mechanism of ADHD in children with SPD. We found delayed/disrupted white matter microstructure in SPD+ADHD compared to SPD-ADHD, especially in males, based on lower FA and especially lower NDI values. This is important because SPD is a common yet understudied disorder that can affect up to one in six children. In our cohort, 43% of individuals with SPD also met research criteria for ADHD, which replicates what has been shown in a previous and unrelated cohort (Anguera et al., 2017), and is up to ten-fold higher than the rate of ADHD in the general population of children (Ben-Sasson et al., 2009; Koziol and Budding, 2012).

SPD refers to a clinical deficit in the ability to modulate, discriminate, or create an organized response to sensory information. As a result, such children may suffer from emotional, social, and educational problems, including anxiety, aggression, inattention, poor self-concept, and academic failure (Miller et al., 2009). The most effective treatment for SPD is sensory integration. A recent randomized clinical trial has proven the effectiveness of occupational therapy with a sensory integration approach compared to placebo in improving the social and cognitive deficits of children with SPD (Miller et al., 2007). Additionally, digital cognitive training of SPD+ADHD subjects has shown sustained benefits for attention at three-year follow up (Jurigova et al., 2021). However, a timely diagnosis and proper therapy are keys to successful treatment, necessitating the development of new objective biomarkers for diagnosis of SPD with deeper understanding of the co-occurring challenges including attention, fine motor control, and emotional regulation. Furthermore, there is a concern that SPD is not a recognized or singular disorder and thus may not warrant study. While we agree that SPD is an umbrella term used to include multiple sensory domains and dimensions, as sensory input and processing with the additional component cognitive control being the basis for thought and action, the dedicated understanding of all aspects of the neural architecture underlying typical and divergent performance is of critical importance. Children with SPD do not usually exhibit morphological abnormalities on structural MRI; however, prior work has demonstrated they have strikingly decreased white matter microstructural integrity in the form of lower FA values on DTI compared to typically developing children, particularly in the posterior cerebral regions that subserve sensory processing and integration (Owen et al., 2013). With DTI findings codifying differences in children with SPD and emerging reports of genetic etiologies (Chang et al., 2015a,b; Mulligan et al., 2019), it is clear that whatever term we use to describe it, be it dysfunction, disorder, or condition, sensory processing needs further study. By pairing phenotyping with advanced neuroimaging, we can further our understanding of SPD as a “brain-based” condition that can be understood and improved with treatment.

Volumetric analyses of children with ADHD, but not screened for SPD, have shown decreased volume in the prefrontal cortex, right basal ganglia (globus pallidus, putamen, caudate), cerebellum, and corpus callosum (Ellison-Wright et al., 2008; Frodl and Skokauskas, 2012; Dougherty et al., 2016). White matter abnormalities in ADHD have been studied using DTI, although showing heterogeneous results with a lack of consensus among studies. A recent investigation found that FA values in the inferior frontal-striatal tract, inferior frontal-occipital fasciculus, SLF, and corpus callosum are negatively correlated with the ADHD polygenic risk score and longer screen time utilization (Yang et al., 2022). A meta-analysis of DTI TBSS studies suggests that the fronto-striatal-cerebellar circuit plays a crucial role in the pathophysiology of ADHD (Chen et al., 2016).

The benefits of NODDI over DTI were robust in our study, where NODDI uncovered microstructural differences in more white matter tracts compared to DTI in SPD+ADHD relative to SPD-ADHD. Non-gaussian white matter microstructure imaging in ADHD is sparse. One study has used Diffusion Kurtosis Imaging (DKI) and found that patients with isolated ADHD had significantly greater WM microstructural complexity than typically developing controls in the bilateral frontal and parietal lobes, insula, corpus callosum, and right external and internal capsules (Helpern et al., 2011). However, as a statistical model of diffusion akin to DTI, DKI is non-specific relative to NODDI and its three-compartment biophysical tissue model (Kamagata et al., 2016). There has been one prior paper using multi-shell diffusion MRI with biophysical modeling to study ADHD that demonstrated decreased dendrite density and volume in certain frontal lobe white matter fiber tracts that subserve the attention function and executive control system (Wu et al., 2020).

Our voxelwise and tract-level statistical analyses with multiple comparisons correction found that boys with SPD+ADHD have decreased NDI in many projection tracts, including the posterior corona radiata, which subserves higher-order sensory functions including multisensory integration, the retrolenticular limb of the internal capsule, which contains the somatosensory and auditory radiations, as well as the posterior limb of the internal capsule and the cerebral peduncles, which contains corticobulbar and corticospinal projection fibers. In addition, commissural fibers of the splenium of the corpus callosum also exhibit low NDI in SPD+ADHD in boys, therefore involving interhemispheric sensory connectivity. Given that WM NDI increases with age during childhood (Chang et al., 2015a,b), this posterior WM tract maturational delay/disruption suggests an association between the degree of sensory tract pathology and the emergence of comorbid ADHD, rather than ADHD being an entirely separate dimension of SPD. These NDI differences between groups cannot be explained by normal brain development, since the male SPD+ADHD and SPD-ADHD cohorts have almost identical mean ages and standard deviations (Table 2).

Furthermore, the bilateral anterior limbs of the internal capsule also show reduced NDI in SPD+ADHD males, implicating prefrontal projection fibers including fronto-striatal connectivity that has been previously implicated in ADHD by a meta-analysis of DTI studies using TBSS (Chen et al., 2016). The maturation of frontal attentional feedback to posterior sensory systems requires normal sensory development. Frontal pathways governing executive function and impulse control lag behind sensory pathway development and their maturation extends well into adolescence. Hence, the hypothesis of abnormal sensory pathway development causing attentional and executive control dysfunction is compelling and merits further investigation.

In addition to these white matter microstructural differences between SPD+ADHD and SPD-ADHD, we found a sexual dimorphism that suggests at least partially different underpinnings in males versus females. There were much stronger NODDI group differences in boys than in girls. Hypotheses about the mechanism of ADHD in girls with SPD require further study in a larger cohort, given the relatively low rates of both ADHD and SPD in girls compared to boys.

### Limitations and future directions

The main limitation of this initial study of ADHD in the SPD population is the modest sample size. Our cohort has a larger number of males compared to females, which mirrors the known sex distributions of both SPD and ADHD. Hence the female subgroup was underpowered to detect small or medium effect sizes (Owen et al., 2013). Second, both SPD and ADHD are heterogeneous disorders with various phenotypic subgroups that might have unique white matter microstructures. Larger-scale studies will be needed to evaluate the subtypes of both SPD (visual, auditory, tactile, etc.) and ADHD (impulsive/hyperactive, inattentive, and combined). Included in the limitations are the strong assumptions made by the NODDI model. If these assumptions are violated, for example by intra-axonal diffusivity changes between SPD+ADHD and SPD-ADHD, this will be transferred to changes in ODI and NDI. While there would still be empirical relevance, the interpretation of the NDI and ODI results in terms of axonal water fraction and orientation dispersion, respectively, would no longer necessarily be correct. Future work is needed to replicate and generalize these results to other higher-order models of the diffusion MRI signal, including representations such as DKI and q-space imaging (Yeh et al., 2010), as well as other biophysically-inspired models such as white matter tract integrity [WMTI; (Jelescu et al., 2015)] and the recently proposed Standard Model that has minimal constraints (Coelho et al., 2022). This would help determine whether the advantages of multi-shell dMRI over DTI stem from the greater diffusion-weighting strength and/or from the more specific modeling of white matter microstructure.

## Conclusion

Lower FA and especially lower NDI in males with SPD+ADHD compared to SPD-ADHD suggest more delayed and/or disrupted white matter microstructure of the former group. Multi-shell diffusion imaging with NODDI was more sensitive than traditional DTI for detecting these group differences. The posterior distribution of most of the observed white matter differences suggests that a greater degree of sensory tract microstructural disruption is associated with co-morbid ADHD.

## Data availability statement

The datasets presented in this study can be found in online repositories. The names of the repository/repositories and accession number(s) can be found at: NIH Data Archive (https://nda.nih.gov) Accession Number:4095004.

## Ethics statement

The studies involving human participants were reviewed and approved by Committe on Human Research, University of California, San Francisco. Written informed consent to participate in this study was provided by the participants’ legal guardian/next of kin.

## Author contributions

EM and PM: conception and design of the study. IM, ML, MR, JW-J, JX, LC, IB, and SP: acquisition and analysis of the data. IM, JW-J, JX, LC, ML, SP, EM, and PM: drafting significant portion of the manuscript and figures. All authors contributed to the article and approved the submitted version.

## Funding

This work was funded by an American Society of Neuroradiology (ASNR) Scholar Award to IM and a National Institutes of Health (NIH) R01 MH116950 grant to PM and EM.

## Conflict of interest

ML, MR, and EM were employed by Cortica Healthcare during the conduct of this work.

The remaining authors declare that the research was conducted in the absence of any commercial or financial relationships that could be construed as a potential conflict of interest.

## Publisher’s note

All claims expressed in this article are solely those of the authors and do not necessarily represent those of their affiliated organizations, or those of the publisher, the editors and the reviewers. Any product that may be evaluated in this article, or claim that may be made by its manufacturer, is not guaranteed or endorsed by the publisher.
