# Supplementary figures and images for: Neurite orientation dispersion and density imaging of white matter microstructure in sensory processing dysfunction with versus without comorbid ADHD
Source: Front Neurosci. 2023 Jul 10;17:1136424. doi: 10.3389/fnins.2023.1136424 (PMC10363610; doi:10.3389/fnins.2023.1136424)

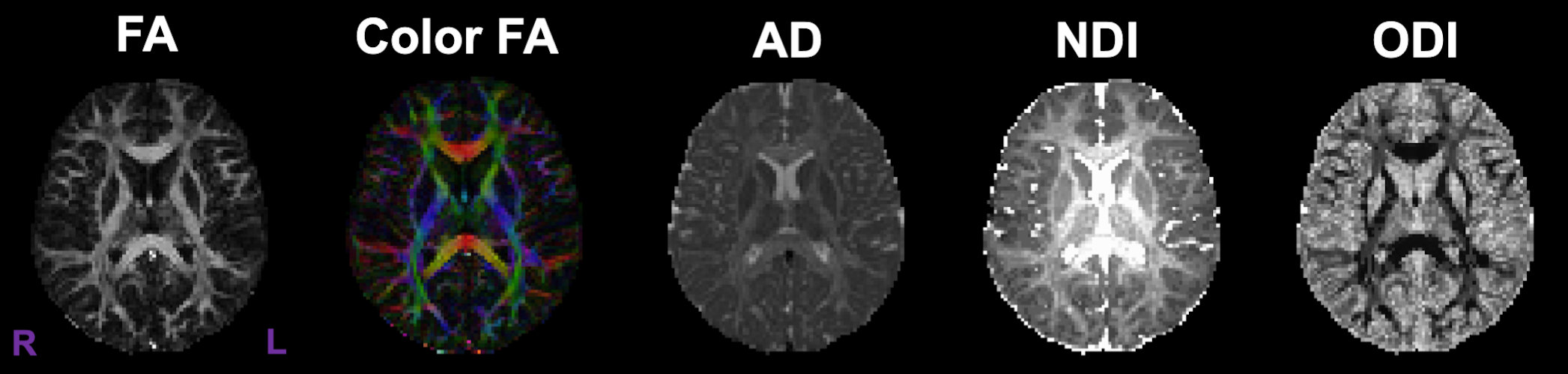

Supplement: Supplementary file 1 [file Image_1.TIF]
